# Honey bee predisposition of resistance to ubiquitous mite infestations

**DOI:** 10.1038/s41598-019-44254-8

**Published:** 2019-05-24

**Authors:** Bart J. G. Broeckx, Lina De Smet, Tjeerd Blacquière, Kevin Maebe, Mikalaï Khalenkow, Mario Van Poucke, Bjorn Dahle, Peter Neumann, Kim Bach Nguyen, Guy Smagghe, Dieter Deforce, Filip Van Nieuwerburgh, Luc Peelman, Dirk C. de Graaf

**Affiliations:** 1 Department of Nutrition, Genetics and Ethology, Ghent University, B-9820 Merelbeke, Belgium; 2 Department of Biochemistry and Microbiology, Ghent University, B-9000 Ghent, Belgium; 3 Wageningen University & Research, 6708PB Wageningen, The Netherlands; 4 Department of Plants and Crops, Ghent University, B-9000 Ghent, Belgium; 5 Honeybee Valley, Ghent University, B-9000 Ghent, Belgium; 6 Norwegian Beekeepers Association, NO-2040 Kløfta, Norway; 7 Department of Animal and Apicultural Sciences, Norwegian University of Science and Technology, NO-1432 Ås, Norway; 8 Institute of Bee Health, University of Bern, 3097 Bern, Switzerland; 9 Department of Functional and Evolutionary Entomology, Gembloux Agro-Bio Tech, University of Liege, B-5030 Gembloux, Belgium; 10 Department of Pharmaceutics, Ghent University, B-9000 Ghent, Belgium

**Keywords:** Agricultural genetics, Ecological genetics

## Abstract

Host-parasite co-evolution history is lacking when parasites switch to novel hosts. This was the case for Western honey bees (*Apis mellifera*) when the ectoparasitic mite, *Varroa destructor*, switched hosts from Eastern honey bees (*Apis cerana*). This mite has since become the most severe biological threat to *A. mellifera* worldwide. However, some *A. mellifera* populations are known to survive infestations, largely by suppressing mite population growth. One known mechanism is suppressed mite reproduction (SMR), but the underlying genetics are poorly understood. Here, we take advantage of haploid drones, originating from one queen from the Netherlands that developed *Varroa*-resistance, whole exome sequencing and elastic-net regression to identify genetic variants associated with SMR in resistant honeybees. An eight variants model predicted 88% of the phenotypes correctly and identified six risk and two protective variants. Reproducing and non-reproducing mites could not be distinguished using DNA microsatellites, which is in agreement with the hypothesis that it is not the parasite but the host that adapted itself. Our results suggest that the brood pheromone-dependent mite oogenesis is disrupted in resistant hosts. The identified genetic markers have a considerable potential to contribute to a sustainable global apiculture.

## Introduction

The ubiquitous ectoparasitic mite *Varroa destructor*, an invasive species from Asia, is the most important biological driver of global losses of honey bee, *Apis mellifera*, colonies^1^. *A. mellifera* is not the original host however as this mite occurred first in the Asian honey bee, *Apis cerana*^1^. Whereas *A. cerana* and *V. destructor* have a long history of co-evolution, this is not the case for *A. mellifera*^2^. This short period of co-evolution has left *A. mellifera* vulnerable^2^. To avoid colony collapse, treatment with acaricides seemed the best option and this is what mite control strategies have relied on for more than five decades. However, scattered observations in affected regions show that untreated honey bee colonies can survive mite infestations^3–5^. This prompted several initiatives aimed at breeding these *V. destructor*-tolerant or -resistant bees (VR)^6–8^. ‘Tolerance’ is a defence strategy whereby the host can limit the harm caused by a given parasite burden, whereas ‘resistance’ refers to the ability of the host to limit the actual parasite burden itself^9^. Both social and individual traits have been implicated in the defence against the *V. destructor*-mite. *Varroa* sensitive hygiene (VSH) is a social behaviour trait that consists of three components (detection, opening and removal of infested and damaged pupae) that are each inherited independently^9^ and is expressed by the adult worker honey bees. It was formerly called ‘suppressed mite reproduction’ (SMR) as reproductive mites were eliminated by hygienic behaviour. However, the term SMR is no longer used for the social, hygienic behaviour as true SMR has been observed in several populations. As such, SMR is currently defined as a trait where mites fail to produce offspring in honey bee pupae by a not yet identified mechanism. This phenomenon of true resistance emerged independently from natural selection on the island Gotland (Sweden^5^) and in Avignon (France^4^), and there are subtle differences between these two distinct bee populations in how they succeed in reducing the reproductive success of the *V. destructor* mites. In more detail, these resistance phenotypes are delayed oviposition and actual infertility of the mother mites, respectively^10^. SMR can be expressed both in worker and drone brood, which are called worker and drone brood resistance (DBR), respectively. Whereas both types of resistance are beneficial, drone brood has a longer pupation time relative to worker brood, which gives the *V. destructor* parasite more time to reproduce. As a consequence, any disturbance of mite reproduction in drone brood will affect mite population dynamics significantly^9^.

Given the burden on the honey bee population, unravelling the genetic architecture of DBR has recently gained much interest for several reasons^11^. Firstly, identifying the exact molecular mechanisms might lead to a better understanding of the host pathogen interaction and new eradication strategies. Secondly, the identification of genetic markers associated with the phenotype can also immediately be used to selectively breed colonies that are more resistant.

Here, we investigate the possibility to identify DBR-associated markers by comparing VR and *V. destructor*-sensitive drones (VS). One of the difficulties when performing these kind of case-control studies is however the risk of spurious associations that might arise due to population stratification^12,13^. An ideal solution is having access to one population where all cases and controls are equally related. As within a colony, it is one queen that gives rise to many equally related drones, we aimed to create one colony containing both VR and VS drones at equal frequencies by selective breeding.

When successful, both reproducing and non-reproducing mites will be identified in that colony. While non-reproducing mites can be a true consequence of host adaptation, which is the desired mechanism, non-reproduction might however also be a consequence of these mites being a distinct subgroup of *V. destructor* parasites lacking the ability to reproduce. While we hypothesize that non-reproduction is a consequence of host adaptation, this will first be investigated using microsatellites of both reproducing and non-reproducing *V. destructor* mites. Upon confirmation of DBR, the identification of markers can be initiated.

Previously, attempts to identify quantitative trait loci all relied on coarse (with at most 3000 markers) and indirect (i.e. linkage disequilibrium-based) mapping strategies, often with inconsistent results^14–16^. The downside of these indirect approaches is that they always require further steps downstream (e.g. fine mapping, candidate gene sequencing). Contrary to this approach, whole exome sequencing (WES) has the potential to identify disease-causing mutations directly if they fall within target regions. But even if causal mutations reside outside target regions, WES variants discovered during sequencing can be used as tagvariants to identify regions associated with the phenotype^17^. Based on previous experiences^18,19^, we hypothesize that WES will allow mapping at a far higher resolution relative to previous approaches. As such, we aimed to develop and evaluate the first WES design for the honey bee and immediately assessed its performance to identify variants associated with DBR.

While WES is a technological step forward, association studies, including the coarse mapping studies already performed for DBR, still often use single variant association tests in their data analysis. Given the phenotype and in line with previous studies, a multifactorial, complex inheritance mode was however expected for DBR^14–16^. As this involves the combination of an (unknown) number of loci and single-marker tests only analyze the marginal effect between a phenotype and a variant, these single-marker tests ignore important information when multiple variants are associated with complex phenotypes. In this study, we use a novel analysis method, called elastic-net penalized regression, that allows joint modelling of variants and hypothesize it outperforms the standardly used single-marker tests.

Finally, to allow a broad use of markers identified in one single colony, it is important that these markers segregate in the general population. We investigate this hypothesis by performing a population screening.

## Results

### Creating a hybrid VR/VS colony

Virgin queens from honey bee stocks selected for VR were selected from different locations in Europe. Three populations became VR after several years being left untreated against the *V. destructor*-mite in the Netherlands (Amsterdam Water Dunes^20^), France (Toulouse^21^) and Norway (Østlandet Region^22^). From Belgium, virgin sister queens from a breeding queen with the highest recorded *Varroa*-index for Belgium in 2014 were selected. This *Varroa*-index combines measurements of the *V. destructor* population dynamics and hygienic behaviour (further explained in materials and methods)^8^.

**Figure 1:**
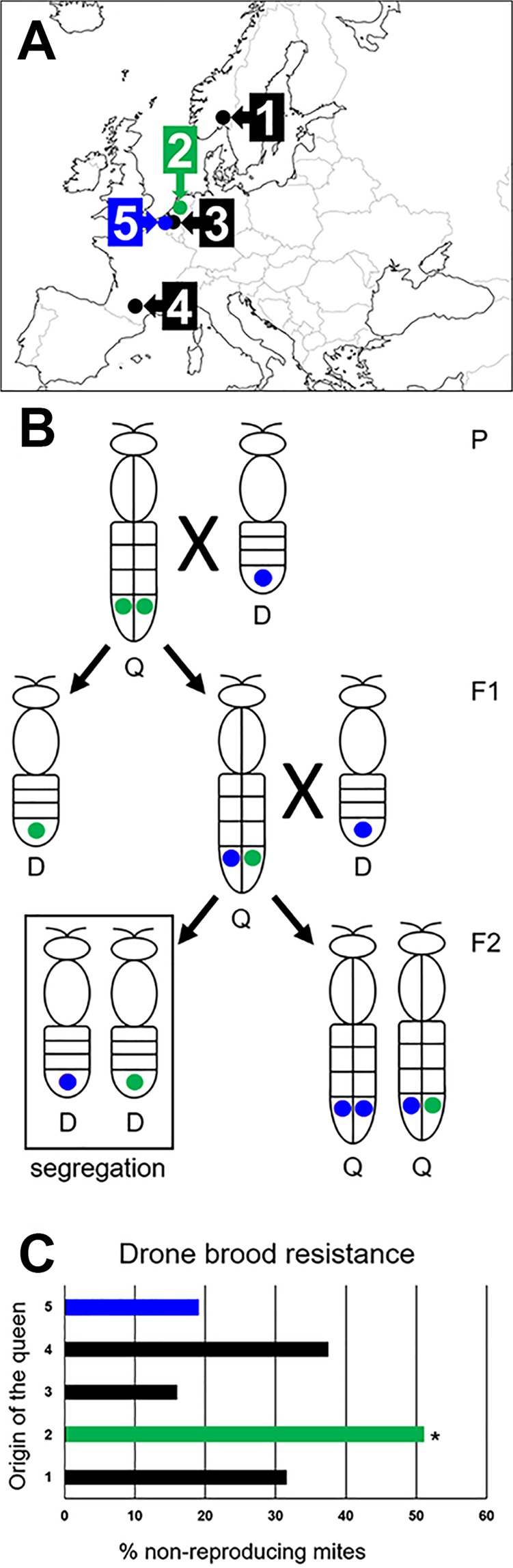
Overview of the breeding experiment and phenotypical screening. (**A**) Origin of the honey bee populations used in this study. Numbers 1–4 were selected for *Varroa destructor*-resistance; number 5 was the *Varroa*-sensitive control: 1. Østlanded Region, Norway; 2. Amsterdam Water Dunes, The Netherlands; 3. Kapellen, Belgium; 4. Toulouse, France; 5. Ghent, Belgium. (**B**) Crossing scheme to obtain hybrid *Varroa-*resistance/*Varroa-*sensitive colonies. For one hypothetical locus associated with the phenotype, the allele associated with drone brood resistance (DBR) is coloured green, while the opposite (undesired) allele is coloured blue. The resulting F2 drones were phenotyped for DBR. In reality, the situation is more complex as several variants, as mentioned in Table 1, were found to be associated with DBR. P = parentalis; F = filialis; Q = queen; D = drone. (**C**) Outcome of the screening for the DBR phenotype in the different crossed populations. This graph depicts the percentage of non-reproducing mites for each of the 5 colonies. To assess whether this DBR phenotype segregated at significantly different frequencies relative to the control population (blue), a Fisher exact test was performed. Only for the Amsterdam Water Dunes colony (green), this result was significant at the 0.01 level. Due to the very high DBR prevalence, the Amsterdam Water Dunes bee colony was used in the subsequent exome sequencing. Other p-values can be found in Table S1. *P = 0.01.

These queens were next crossed twice with local VS drones (Fig. 1). In more detail, this implies single VS drone artificial insemination of VR queens in the parental generation (P), leading to hybrid VR/VS queens in the first filial generation (F1). These F1 hybrid queens carrying both VR- and VS-associated alleles were then mated naturally with local VS drones. As only the queens give rise to drones in the subsequent generation, this allows segregation of both VR and VS alleles in the second filial (F2) haploid drone brood generation (Fig. 1). To eliminate the effect of environment as much as possible, all F2 colonies were reared at the same location. All five populations (the four VR hybrids and the control strain) were successfully bred and maintained.

### Screening for the DBR phenotype

Individual host brood cells were opened at the adequate age and assessed for *V. destructor*-mite infestation (as described in more detail in materials and methods). This screening revealed that the percentage of non-reproducing mites in these four hybrid VR populations was 51.0% (the Netherlands), 37.5% (France), 31.6% (Norway) and 14.0% (Belgium), respectively, whereas this percentage was 19.0% in a local VS control strain (Table S1). The colony from the Netherlands had the highest frequency non-reproducing mites. This was also the only colony where this frequency was significantly higher relative to the local control strain (P = 0.01), which indicates that the DBR phenotype from this colony can be transmitted to subsequent generations, i.e. it is suggestive for a genetically-based resistance mechanism. Furthermore, since the queens of the F1 generation were VR/VS hybrids, the outcome of F2 DBR typing for this colony was even unexpectedly high (expected maximum score was 50%) and corresponds to what can be expected in a Mendelian mode of inheritance, which makes it ideal for subsequent association studies. As such, this Dutch colony was selected and used further downstream in the WES part of this study.

### *Varroa destructor*-mite genotyping

**Figure 2:**
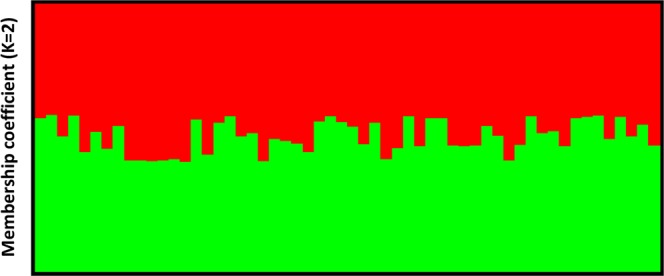
Bayesian clustering of the *Varroa destructor*-mites originating from eight beehives. Each vertical line stands for an individual specimen, while the colors are indicative for the proportion a specimen belongs to a certain group.

In order to verify whether the observed DBR phenomenon is due to host adaptations and not a parasite-mediated effect, 56 mother mite specimens originating from drone brood cells with non-reproducing mites (=DBR phenotype) or with reproducing mites were genotyped at 13 microsatellite loci (Table S2)^23^. All 13 loci amplified successfully, which led to a total of 23 different alleles, varying from one to eight per locus (Table S2). Per colony, the effective number of alleles ranged from 1.145 to 1.502 (Table S3). The observed heterozygosity *H*_O_ values were very low ranging from 0.000 to 0.077. The mean expected heterozygosity *H*_E_ was 0.130, with values ranging from 0.075 to 0.186 within each colony. As expected, the inbreeding coefficients were very high (mean: 0.788) demonstrating clear evidence of inbreeding between the *V. destructor*-mites within a colony (Table S3). Furthermore, no significant differences in these genetic parameters were found between reproducing and non-reproducing mites from a colony (Paired *t*-tests, *df* = 7, *P* > 0.05), nor when comparing all reproducing and non-reproducing mites (Independent *t*-tests, *df* = 6, *P* > 0.05). The Evanno method identified ΔK = 2, which implies that K (or the number of populations) equals two or one (as ΔK cannot be used to distinguish those two values)^24^. Based on the results from the individual mites, K was set to one as each mite belonged for approximately 50% to both groups (Fig. 2). This implies that these mites could not be genetically distinguished from each other. Based on these results, the observed DBR seems to represent the real and desired genetic resistance phenotype and is not a consequence of differences between mites that might mimic it.

### Whole exome sequencing

To identify genetic variants associated with the DBR phenotype, a WES design targeting all exons of the *A. mellifera* genome (Amel.4.5)^25^ was developed. This design contained 26,184,643 base pairs (bp) divided over 81,571 regions. Sixty-four drones (32 DBR positive; 32 DBR negative), originating from the colony from the Netherlands (i.e. from the colony that differed significantly from the local control strain in terms of the number of non-reproducing mites), were selected and Illumina sequenced. In these drones, a median coverage of 64.5x was obtained, while >97% of the 26 Mb target bp pairs were covered at ≥10x (Fig. S2; Table S4). In terms of regions, the median number of regions that were entirely sequenced at ≥10x was 91.3% (Fig. S3; Table S4). The sequencing reproducibility was high, with close to 75% of the regions entirely sequenced in all samples. Similar to other WES designs, we found that sequencing performance was inversely correlated with target region length and low complexity/highly repetitive nucleotide composition, whereas GC nucleotide composition of a target region and quality of the target region (reflected by the proportion of ambiguous nucleotides in the target region) affected sequencing performance far less (Fig. S4; Table S5)^19^. The aforementioned results demonstrate that the novel WES design outperforms several WES designs in other species^18,19^.

Next, we looked at the number of variants that can be used for the association study. Overall, more than 140,000 variants with a call rate of at least 99% were discovered inside the exons. Both in terms of the number of markers (50 times higher), as with respect to the resolution (the median and mean distance between markers were 71 bp (first quartile: 18 bp – third quartile: 342 bp) and 1562 bp, respectively (Fig. S5)), this is by far the most dense study on DBR conducted up until now^14–16^.

### Single-marker tests and elastic-net penalized regression

Whereas single-marker tests are most commonly used, previous studies on this phenotype have shown that these tests often lack the necessary power to obtain significant results^15,16^. The same result was observed here as no single variant reached genome-wide significance (Fig. S6). This was no surprise as for the proposed complex inheritance of the phenotype, single-marker tests are far from ideal as these tests only analyze the effect of an individual variant on a phenotype, while ignoring all the others^26–29^.

A potential solution is to model all variants jointly. In genetic studies, including the one presented here, there are however additional complications that have to be taken into account^26–30^. Firstly, the number of variants (=parameters *p*) often exceed the sample size *n* by far (often referred to as “the small *n*, large *p*” problem). Secondly, both due to the high dimensionality (i.e. the large number of variants) as the actual physical linkage between variants, different variants can be correlated with each other, leading to the multicollinearity problem.

Where standard multiple regression cannot deal with these issues, the so-called elastic-net penalized regression that combines the strengths of LASSO in terms of parameter selection and ridge regression to deal with correlated variants, solves all these problems^26–28^. Based on the combination of cross-validation and stringent cut-offs, elastic-nets have shown to control the number of false positives (often perfect, i.e. no single false positive result) while at the same time identifying a large number of true associated variants^28^. In this study, the same strict methodology was used, which resulted in eight variants (eight SNPs) in seven different genes that were found to be associated with the DBR phenotype (Table 1). From these eight mutations, two were missense, altering the amino-acid composition, while the remaining six were silent mutations. Altogether, this eight variants model predicted 88% of the initial sixty-four phenotypes correctly (56/64) (Table 2) and identified six risk (i.e. associated with a lower resistance to *V. destructor* mites) and two protective (i.e. associated with a higher resistance to *V. destructor* mites) variants.

**Table 1:** Variant description together with the effect size (β) and allelic frequency analysis giving the number of colonies where the mutations were found (from the total of 46 colonies that were sampled).

Scaffold	Location	Gene	Gene name	Variant	RNA level	Protein level	Effect	β	% Present (n)
Group1.41	909712	GB54921	mucin-12 isoform X1	T > C	GB54921-RA:r.144 A>G	GB54921-PA:p.A47 = (or Ala47=)	Silent	0.23	91% (42/46)
Group1.41	909762	GB54921	mucin-12 isoform X1	C > T	GB54921-RA:r.94 G>A	GB54921-PA:p.V32I (or Val32Ile)	Missense	0.22	89% (41/46)
Group10.23	545027	GB48382	solute carrier family 22 member 21	C > T	GB48382-RA:r.987 G>A	GB48382-PA:p.A328 = (or Ala328=)	Silent	−0.06	78% (36/46)
Group15.14	757132	GB50526	sodium-coupled monocarboxylate transporter 1	C > T	GB50526-RA:r.1662G>A	GB50526-PA:p.P554 = (or Pro554=)	Silent	−0.15	9% (4/46)
Group15.19	133198	GB50114	dynein beta chain, ciliary	T > C	GB50114-RA:r.1662A>G	GB50114-PA:p.P3167 = (or Pro3167=)	Silent	0.26	73% (34/46)
Group3.15	494900	GB47018	uncharacterized protein LOC724886 isoform X2	G > A	GB47018-RA:r.1824C>U	GB47018-PA:p.L608 = (or Leu608=)	Silent	0.30	87% (40/46)
Group9.12	805359	GB53345	uncharacterized protein LOC100578770	T > C	GB53345-RA:r.37 A>GG	GB53345-PA:p.M13V (or Met13Val)	Missense	0.02	98%(45/46)
Group9.12	888963	GB53340	spectrin beta chain isoform X1	A > C	GB53340-RA:r.4143U>G	GB53340-PA:p.V1381 = (or Val1381=)	Silent	0.09	96% (44/46)

The intercept of the model equals −0.39 (model settings: α = 0.9, λ = 0.18).

**Table 2:** Contingency table comparing the predicted phenotypes based on the eight variant model (“Model”) relative to the observed phenotypes (“Truth”) of the 64 drones used in the whole exome sequencing experiment.

Model	Truth	
	Control	Affected
Control	29	5
Affected	3	27

Fifty-six out of 64 drones were correctly classified (88%).

So far, previous (coarse) mapping studies on DBR in the Swedish bee population identified loci at chromosome four, seven and nine^15^ and the chromosomes two, three and fifteen, respectively^16^. In our study, SNPs at chromosome nine and chromosome fifteen were retained in the final model. While we believe this substantiates the results found in this study, at the same time, it is clear that, in line with the (partially differing) DBR phenotype, both populations (the Swedish one from the previous studies versus our Dutch population from this study) also developed a unique way of dealing with the same parasite.

### Allelic frequency analysis in the Belgian honey bee population

As a final step, a stratified sampling strategy was used to evaluate the allelic frequencies of the previously identified associated variants in the general bee population in Belgium. Sanger sequencing, each time two bees per colony for a total of 46 colonies, revealed a widespread distribution of all variants throughout the bee colonies (Table 1). This indicates that these variants are not colony specific, which facilitates their use in centrally coordinated population-wide selection programs. Furthermore, on average, the risk mutations were found in more colonies (89%; 41/46 colonies) relative to the protective mutations (43%; 20/46 colonies), which is no surprise given the widespread *V. destructor-*sensitivity in the Belgian honey bee population. Finally, in agreement with the expectations for complex diseases, there was a clear negative correlation (Spearman’s correlation coefficient = −0.38) between the prevalence of the mutations in the population and the effect size (i.e. the absolute magnitude of the β coefficients of the different variants as depicted in Table 1)^31^.

### Hypothesized biological involvement of the identified genes

As mentioned and in agreement with literature, the DBR phenotype was expected to segregate in a complex manner, requiring the involvement of several genes^14–16^. In our study, variants in seven genes (i.e. mucin-12 isoform X1, solute carrier family 22 member 22, sodium-coupled monocarboxylate transporter-1, dynein beta chain, spectrin beta chain isoform X1 and two uncharacterized proteins; Table 1) were found and both synonymous and non-synonymous variants were identified. It goes without saying that these variants can both be actual phenotype-causing variants or might just be markers associated with it. While traditionally non-synonymous mutations are often focused on, recent studies, however, strongly support the potential role that synonymous mutations on (complex) phenotypes might have^32–34^. In addition, the involvement of the dynein beta chain in the DBR phenotype was remarkable, as it represents a cytoskeletal motor protein involved in intracellular retrograde transport along the microtubules of eukaryotic cilia and flagella^35^. Among insects, cilia/flagella are present in the sperm tail (flagellar) and in mechano- and chemosensory neurons (ciliary) only^36,37^. Moreover, the ciliary form of dynein that we identified here can exclusively be related with sensory functions of the honey bee antenna, most probably through its olfactory neurons.

In honey bees, brood care is secured through a few behavioural sequences (feeding, brood cell capping, thermoregulation) that are initiated by brood pheromones^38^. The sensing of these cuticular hydrocarbon pheromones occurs on the insects’ antennae, more in particularly by specialized olfactory sensillae^39^. It has been demonstrated that the *V. destructor*-mite synchronizes its reproduction with the ontogenic development of the honeybee larvae and that the mite’s oogenesis is triggered by volatiles of the larval cuticle^40,41^. We hypothesize that the DBR phenotype was obtained by two phenomenons. Firstly, the variant of the dynein beta chain found in DBR positive bee colonies causes a better pheromone sensing by an improved intracellular transport in the olfactory neurons. Secondly, the other variants cause a diminished production of the cuticular hydrocarbon brood pheromones by a reduced general or tissue-targeted (integumentum) metabolism. Here, the different transporters might play an important role^42^. Consequently, the pheromone release falls to a level that is no longer able to initiate oogenesis in the mite, although it permits normal chemical communication - and thus brood care - by the adult bees. The hypothesis links several of the genes from which a variant is associated with the DBR phenotype, but so far any experimental evidence is lacking. However, a similar strong involvement of the olfactory sensing has been demonstrated by transcriptome studies^43–45^ of the VSH phenotype that renders bees resistant to *V. destructor*-mite infestations by hygienic behaviour. In that case again intracellular transport and vesicle trafficking in the antennae is one of the underlying biological mechanisms^44,46^. In addition, in the Gotland VR bees a glucose-methanol-choline oxidoreductase was found to be putatively involved in changing volatiles emitted by the bee larvae^14^.

## Discussion

Selection for more resilient bees offers a sustainable solution for the main driver of global honey bee colony losses, i.e. the infestation by the ectoparasitic *V. destructor* mite. Although the voluntary denial of any treatment has already led several times to *V. destructor*-tolerant or -resistant bees, this approach is still difficult for the average beekeeper because of the high losses that may go with it. That is why unravelling how natural selection shapes resilient honey bee populations at both the phenotypic and genetic level, represents a crucial lever to support the classical selection programs through breeding value estimation or marker-assisted selection. The present work developed novel state-of-the-art methodologies for honey bees to discover genetic variants associated with the given trait.

Firstly, we have developed a WES design for the honey bee. In terms of performance, this newly developed WES design excels in comparison with WES designs from other species^19^. Furthermore, relative to for example RESTseq^16,47^ and the 44 K GWAS SNP assay^48^, far more variants are found, which should improve the identification of disease-associated variants, while relative to whole genome sequencing, it is a more cost-efficient approach until sequencing prices drop further. Focusing on exons, it is important to stress that variants outside the target regions are potentially missed, especially for those located at a distance of exonic variants. Overall however, this WES design fulfilled our expectations and we are confident that, for the time being, it will be a valuable tool for further genetic studies of honey bees.

Secondly, we evaluated elastic-net regression and demonstrated that it outperforms single-marker association tests. Combined with WES, it was capable of discovering variants associated with complex traits. To our knowledge, this is the first time that either WES or elastic-nets have been used in the honey bee.

In our approach, we specifically chose to sequence drones derived from one colony for two main reasons. By sequencing equally related bees, the problem of spurious associations due to population stratification was avoided. In addition, while SMR has been reported to have developed naturally in several populations, the underlying mechanism is not entirely the same in every population^10^. As such, there is a high risk that the genetic contributors differ as well, which, in turn, would have reduced the power to detect an association. This is avoided by focusing on one population. A potential downside is that this might lead to a reduced generalizability, i.e. that (at least) some variants associated with the phenotype are unique for that specific population. One option for future studies is thus to redo this experiment by combining drones from different populations.

Also in terms of the population study, we deliberately chose to look for the variants in the Belgian honey bee population. The main reason was a direct evaluation of the potential applicability of the results in that population. Other options that might be pursued in the future are for example looking for the variants in public datasets^49,50^ and other VR populations to investigate how widespread these mutations are.

With respect to the phenotype, the present study describes a very promising trait, i.e. DBR, in an already well-studied resilient honey bee population that was established in the Amsterdam Water Dunes in the Netherlands by natural selection^20^. *V. destructor* has a strong preference for drone brood^51^, making drone brood removal or drone brood ‘cutting’ a common intervention in beekeeping practice and part of a biotechnical *V. destructor* control strategy. As the post-capping developmental stage of drones lasts two days longer than for worker bees (14 days instead of 12 days), more mature *V. destructor*-mites emerge from a drone brood cell. The Cape honey bee (*A. m. capensis*) has an innate resistance against the *V. destructor*-mite by shortening the post-capping developmental time to only 9 days on average^52^. The failure of mite reproduction in the DBR phenotype has a similar effect on the *V. destructor* population dynamics, and thus is an alternative way to get resistance against the mite by avoiding massive mite reproduction in the male brood. Moreover, the costs of this trait for the colony is relatively low when compared to hygienic behaviour, where up to 32.4% of the pupae are removed from the brood^53^. As the identified variants associated with DBR were widespread in the natural bee population, we believe the road to marker assisted selection is open.

## Material and Methods

### Honey bee populations and crossing

Virgin queens from honey bee stocks selected for VR originated from different locations in Europe. From Belgium, we also took virgin sister queens from breeding queen with code 57-584-11612-2012 from Guido Haagdorens, a participant of the Belgian branch of the Beebreed program (Länderinstitut für Bienenkunde Hohen Neuendorf, Germany). This breeding queen was chosen because it gave the highest recorded *Varroa*-index for Belgium in 2014. The Beebreed program relies on breeding value estimation and the *Varroa*-index combines measurements of the *V. destructor* population dynamics and hygienic behaviour^8^. It requires estimations of the daily mite-fall in spring, the size of the phoretic mite population by the powdered sugar method in summer and the clearance of dead pupae by the pin-test^54^.

Six virgin queens of each honey bee stock were crossed twice with local VS drones. In the parental generation, we performed artificial insemination at a queen age of 9–10 days under CO_2_ treatment (this treatment was also given 1 day earlier for 6 min). For each insemination fresh semen was taken from another drone from the same colony (code EXP10) and injected in the queen’s main oviduct (1.5 µl semen + 0.5 µl dilution buffer; dilution buffer contained 0.2 M NaCl, 5 mM glucose monohydrate, 0.67 mM L-lysine, 0.57 mM L-arginine, 0.68 mM L-glutaminic acid, 0.02 M Trisma HCl, 0.03 M Trisma base and 2.5 mg/ml dihydrostreptomycine). Ten to twelve days after introducing the queens in 3-frame hives, we checked for oviposition and nine days later for brood capping. At that moment and when insemination was proven to be successful, we started queen rearing.

From each genetic stock only one colony was chosen for queen rearing. At least four one-day-old larvae from each colony were grafted to artificial queen cells and then transferred to a cell building colony. Once the queen cells were sealed they were transferred to an incubator. The emerging queens were introduced in small mating nuclei colonies, that were treated with oxalic acid (*V. destructor*-treatment) prior to queen introduction. In this first filial generation virgin queens were allowed to mate naturally. Again we checked for oviposition and cell capping. A second mite treament was done just before winter.

In spring, all colonies were moved to the same apiary (campus Sterre) in order to do the testing in the same environment. Drone brood frames were introduced in two colonies per genetic stock. However, only one colony was used for determination of the phenotype.

### Determination of the phenotype

When drone brood cells were capped, the frames were transferred to an incubator and kept at 34 °C. By doing so, we could avoid that our measurements were influenced by hygienic behaviour of the adult bees. Ten days later the drone brood and the *V. destructor*-mites were killed by freezing. This simplifies the determination of the phenotype as mites will no more escape when drone brood cells are opened. We examined drone brood cells for the presence of a mother mite only (non-reproducing) or a mother mite with her progeny (reproducing) in the presence of red-eyed drone pupae. Both drone pupae and mother mites were stored at −80 °C for subsequent WES and *V. destructor* genotyping of selected samples, respectively. A Fisher exact test was used to compare the frequency of the VR and the VS drones in the colonies derived from the VR populations relative to the local VS control population. Significance was set at α ≤ 0.05/4 (Bonferroni correction for multiple testing). This was performed in R v3.4.2 (“Short Summer”).

### *Varroa destructor*-mite genotyping

Fifty-six *V. destructor* specimens were genotyped with 13 microsatellite loci which already have proven to give reliable signals^23^ (Table S2). Ten loci were originally developed for *V. destructor:* seven loci (VD146, VD163, VD001, VD151, VD015, VD112 and VD114) by Cornman *et al*.^55^, and three loci (VD305, VD306 and VD307) by Solignac *et al*.^56^. The final 3 loci (VJ275, VJ294, and VJ292) were developed for *V. jacobsoni*^57^.

Individual DNA was obtained from whole *V. destructor* mites following a Chelex (InstaGene Matrix, BioRad) DNA extraction method as described in Maebe *et al*.^58^. In short, 200 µl Chelex and 10 µl proteinase K was added to an individual mite (sliced with a sterile blade) and incubated for 2 h at 56 °C. After a second incubation step of 15 min at 96 °C, the supernatants of 180 µl (DNA) was frozen in −20 °C until further use.

By multiplex PCR, these 13 microsatellites were amplified by HotstarTAq DNA Polymerase (QIAgen, Belgium) in a total volume of 10 µl. The PCR mix consisted out of 1 µl template DNA, PCR buffer (1x), 0.2 µM dNTP’s, 0.1 µM forward primer, 0.5 µM reverse primer, 0.5 µM different labelled forward M13 primers and 1 unit Taq polymerase. Fluorescent labelling of the PCR products was done using a tailed-primer approach^59^. In this approach a universal M13-primer (=‘tail’, 5′-GAGTTTTCCCAGTCACGAC-3′) was coupled to a VIC, 6-FAM, PET or NED fluorescent label (Table S2). To allow the incorporation of the tail during PCR, the same sequence as the tail was built-in at the 5′-end of all forward primers (see also^60^). Furthermore, the normal annealing temperature of 60 °C was decreased with 2 °C to 58 °C. The other PCR conditions were: a first denaturation step at 95 °C for 15 min, then 30 cycles of 30 s denaturation at 95 °C, 30 s annealing at 58 °C and 30 s extension at 72 °C, followed by a final extension step of 10 min at 72 °C. Visualization of the PCR products was done by capillary electrophoreses on an ABI3730xl sequencer (Applied Biosystems) with help of a 500 LIZ standard (Genescan, Applied Biosystems). The fragments were scored manually with the Peak Scanner v1.0 software (Applied Biosystems). As a quality control, the amplification of 16 randomly chosen samples was repeated.

For the mites originating from the same beehive, several genetic parameters were determined with the program GenAlEx 6.5^61^ including: Nei’s unbiased expected heterozygosity (*H*_E_), the observed heterozygosity (*H*_O_) and the effective number of alleles (*N*e) as parameters of genetic diversity, and also the inbreeding coefficient (*F*is). Paired *t*-tests were performed over all loci to search for differences between reproducing and non-reproducing mites within a colony, while possible differences over all colonies between reproducing and non-reproducing mites were tested with independent *t*-tests. These tests were performed in SPSS.

Hence, the software Structure v2.3.3^62^ was used to perform a Bayesian approach to determine the number of populations (or groups) within the dataset. In this analysis, the number of populations (K) was estimated from 1 to 8, and this was repeated 9 times. Each K-value was calculated with a burn in of 1,000,000 iterations and 500,000 MCMC data collecting steps. Hence, the free online program Structure Harvester v0.6.93^63^, was used to determine the best value of K, and the program Distruct v1.1 software was used for graphical visualization of the population structure.

### Development of exome design

The reference genome (Amel_4.5_scaffolds.fa) and corresponding annotation (amel_OGSv3.2.gff3.gz) were obtained from the Hymenoptera Genome Database^64^. A bed file containing all the exons (including the UTRs) was created with the bedr (v1.0.4) and seqinr (v3.4–5) R-packages. Our design was processed by the Roche Nimblegen custom design group (Madison, USA). Using an SSAHA algorithm, capturing baits were developed based on our design and the reference genome. Design settings for the baits allowed five or fewer single-base insertions, deletions or substitutions between the baits and the genome. Each bait itself was allowed to match up to 20 locations in the genome. Regions under 100 bp were padded to 100 bp to increase capturing efficiency. After approval, the baits were generated and provided as SeqCap Developer Library.

### DNA extraction

Sixty-four pupae (32 coming from a drone brood cell with a non-reproducing mite; 32 with a reproducing mite), all derived from one colony, were subsequently used for DNA extraction. Each pupae was individually homogenized in a total of 1 ml RLTPlus buffer by mechanical agitation in a TissueLyser for 90 s at 30 Hz, in the presence of 4 metal beads and glass beads. One third of the sample was used to isolate DNA and RNA with the ALLPREP DNA/RNA isolation kit from Qiagen following the manufacturers recommendations. The RNA was eluated in 100 µl RNAse free water while the DNA was eluated in 80 µl EB buffer.

### Sample preparation and sequencing

Following DNA extraction, a picogreen assay was performed and 1 μg of every sample was subsequently treated with RNase I (ThermoFisher Scientific). The DNA was next fragmented on a Covaris S2 System in a 130 μl volume (aim: 300 bp fragments, settings: duty cycle: 10%, intensity: 4, cycles per burst: 200, time: 80 s). Depending on the yield after DNA-extraction, between 500 ng and 1 μg of the fragmented DNA was used as input for the library preparation. Samples were end repaired, A-tailed and ligated with TruSeq adapters using the reagents from the KAPA library prep kit according to the manufacturer’s protocol. Size selection was performed on a 2% E-Gel (Invitrogen Life Technologies) (G4010-02), fragments were selected with an insert size around 200–700 bp. Thereafter, the pre-capture LM-PCR was performed on the samples for 8 cycles as prescribed in the SeqCap EZ library protocol. The concentration of each PCR product was determined using Quant-iT™ PicoGreen™ dsDNA Assay Kit (Life Technologies). Sixteen times four samples were equimolarly pooled to obtain a total DNA input of 1250 ng. The pooled library was hybridized for 19 hours and 30 minutes with the baits (SeqCap Developer Library). The hybridized library was washed and the captured and pooled DNA was recovered. After a final amplification (LM-PCR, 13 cycles), the quality of the library was checked using the High Sensitivity DNA chip (Agilent). To check the fold enrichment after capturing, a qPCR is performed as a quality control step before sequencing. The used primers are shown in Table S6. An additional qPCR was performed to determine the quantity of the library to ensure optimal cluster densities. Two times twenty-four samples and one time sixteen samples were sequenced per lane on the NextSeq 500 PE 75 bp.

### Sequencing data-analysis

The reads were aligned to the reference genome (Amel_4.5) using BWA v0.7.15^65^. Duplicate reads were marked with Picard tools v2.1.1. Using the GATK v3.8-0, variants were called according to the GATK Best Practices^66^.

### Variant filtering

From the total list of putative variants, only those were retained that 1/passed the “hard” quality filter suggested from the GATK Best Practices, 2/that had at least 2 different alleles segregating in the population, 3/fell within the target regions and 4/with a call rate of 99%. Filtering was performed with VCFtools v0.1.14 and custom R-scripts^67^. Retrieval of the gene function was done by BLAST searching.

### Single-marker tests and elastic-net penalized regression

For the single marker tests, a Fisher exact test was conducted for each variant. The Bonferroni correction for multiple testing was applied. Elastic-nets penalized regression was next performed with the glmnet v2.0–12 R-package in R v3.4.2 (“Short Summer”)^27^. For the model selection, potential parameters were the 140,151 variants and no covariates were added. To obtain the optimal lambda (i.e. the penalty) and alpha (i.e. the balance between more “lasso”-like and “ridge”-regression like behaviour), a leave-one-out cross-validation was performed for alpha ranging from 0 to 1 (in steps of 0.1). Lambda was set at the stringent MSE+1SE threshold^28^.

### Allelic frequency analysis in the Belgian honey bee population

Worker bees were sampled at the apiaries from breeders involved in the Flemish beekeeping program. Thirty breeders sampled only a single colony; two others sampled four and twelve colonies, respectively. The apiaries are distributed throughout Flanders, the northern part of Belgium. Two individual worker bees from 46 different colonies were sampled from different apiaries. Each single bee was homogenized in 0.5 ml of 100 mM NaCl; 20 mM Tris-HCl, pH 8; 25 mM EDTA, pH 8; 0.5% SDS by mechanical agitation in a TissueLyser for 90 s at 30 Hz, in the presence of metal beads and glass beads. After homogenization 20 µl/ml Proteinase K (20 mg/ml) was added and incubated for 1 hour at 37 °C. First, an equal volume of phenol:chloroform was added and centrifuged at 12,000 *g* for 10 min at 4 °C. The supernatant was then extracted with an equal volume of chloroform:isoamyl alcohol (24:1) and centrifuged at 12,000 *g* for 10 min at 4 °C. The DNA was precipitated using two volumes of ethanol and centrifuged at 12,000 *g* for 30 min at 4 °C. The precipitated DNA was finally washed with 0.5 ml 70% ethanol. The DNA was dissolved in 100 µl DNase/RNase free water.

Primer pairs were designed with Primer-BLAST^68^. Primers were chosen in regions that were free of secondary structures (Mfold) and are listed in Table S7^69^. PCR amplicons were analyzed via agarose gel electrophoresis. Sequencing reactions were performed using the BigDye Terminator v3.1 Cycle Sequencing Kit (Applied Biosystems, Foster City, CA, USA) with the individual PCR primers as sequencing primers and run at Eurofins Genomics (Ebersberg, Germany). Sequence analysis was performed with BioEdit v7.2.6.

The allele frequency was determined at the colony level, followed by a comparison of the average allele frequency for risk and protective alleles and an evaluation of the correlation between the effect size and the allele frequency (Spearman correlation).

## Supplementary information

Supplementary information

## Data Availability

All the data is available upon request.
