# Supplementary material for: Honey bee predisposition of resistance to ubiquitous mite infestations
Source: Sci Rep. 2019 May 24;9:7794. doi: 10.1038/s41598-019-44254-8 (PMC6534585; doi:10.1038/s41598-019-44254-8)
Supplement: Supplementary file 1 — Supplementary information [file 41598_2019_44254_MOESM1_ESM.pdf]

Supporting Information

Honey bee predisposition of resistance to ubiquitous mite infestations

Bart J.G. Broeckx[1,†,*], Lina De Smet[2,†], Tjeerd Blacquière[3], Kevin Maebe[4], Mikalaï Khalenkow[5], Mario Van Poucke[1], Bjorn Dahle[6,7], Peter Neumann[8], Kim Bach Nguyen[9,] Guy Smagghe[4], Dieter Deforce[10], Filip Van Nieuwerburgh[10], Luc Peelman[1,‡] & Dirk C. de Graaf[2,5‡]

[1]Department of Nutrition, Genetics and Ethology, Ghent University, B-9820 Merelbeke, Belgium; [2]Department of Biochemistry and Microbiology, Ghent University, B-9000 Ghent, Belgium; [3]Wageningen University & Research, 6708PB Wageningen, The Netherlands; [4]Department of Plants and Crops, Ghent University, B-9000, Ghent Belgium; [5]Honeybee Valley, Ghent University, B-9000 Ghent, Belgium; [6]Norwegian Beekeepers Association, NO-2040 Kløfta, Norway; [7]Department of Animal and Apicultural Sciences, Norwegian University of Science and Technology, NO-1432 Ås, Norway; [8]Institute of Bee Health, University of Bern, 3097 Bern, Switzerland; [9]Department of Functional and Evolutionary Entomology, Gembloux Agro-Bio Tech, University of Liege, B-5030 Gembloux, Belgium; and [10]Department of Pharmaceutics, Ghent University, B-9000 Ghent, Belgium.

[†,‡] These authors contributed equally to this work.

* Corresponding author: Bart.Broeckx@ugent.be

The supplementary information contains 7 tables and 6 figures.

Fig. S1. ΔK calculated by the Evanno method with the Structure Harvester software[1].

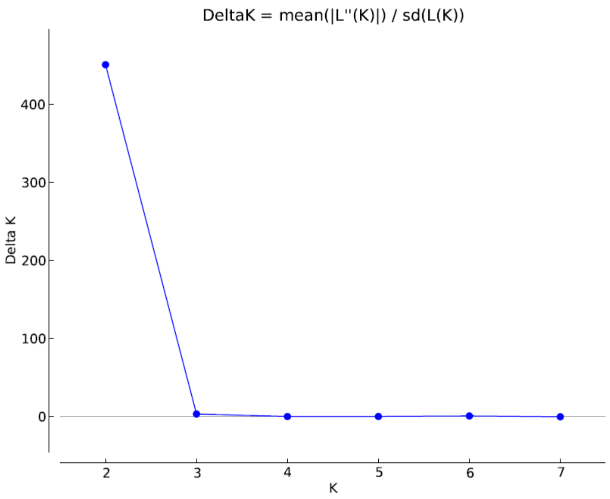

Fig. S2 Relation between realized sequencing depth and the proportion of target base pairs for which this sequencing depth is reached.

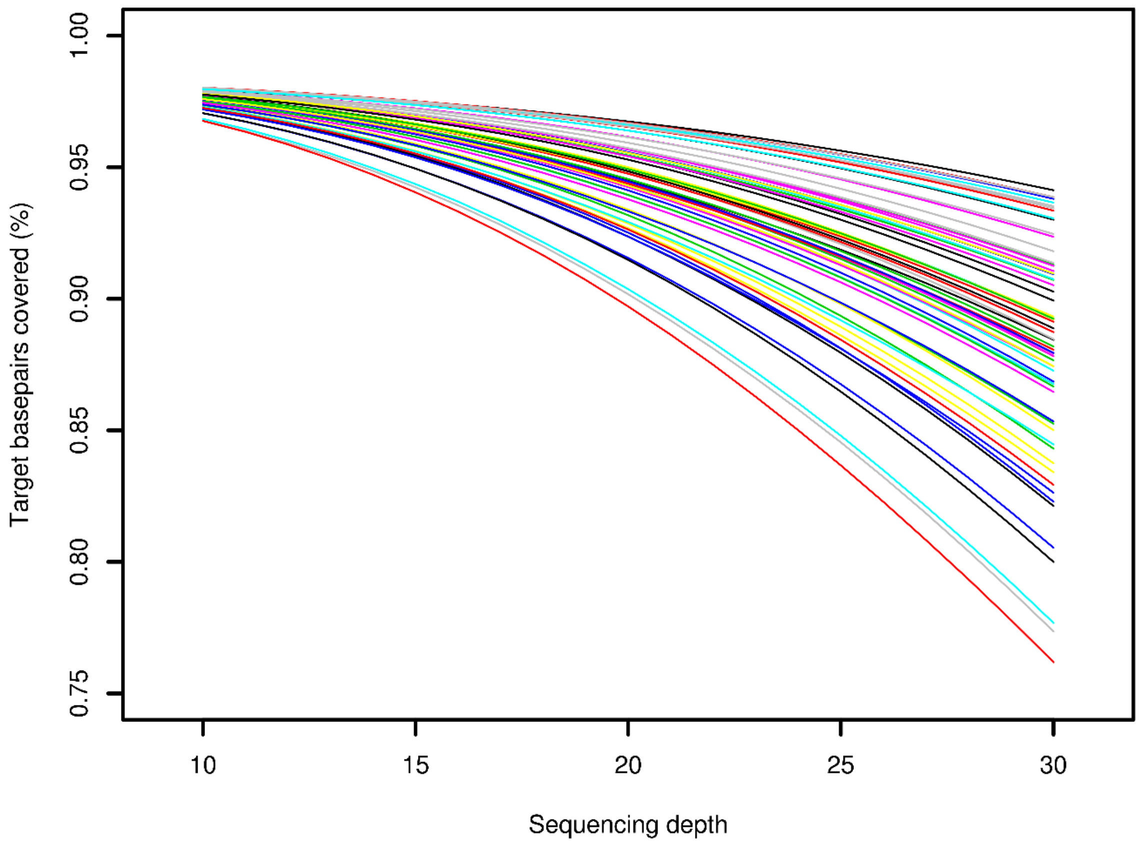

Fig. S3 Relation between realized sequencing depth and the proportion of target regions that were entirely sequenced with at least the specified sequencing depth.

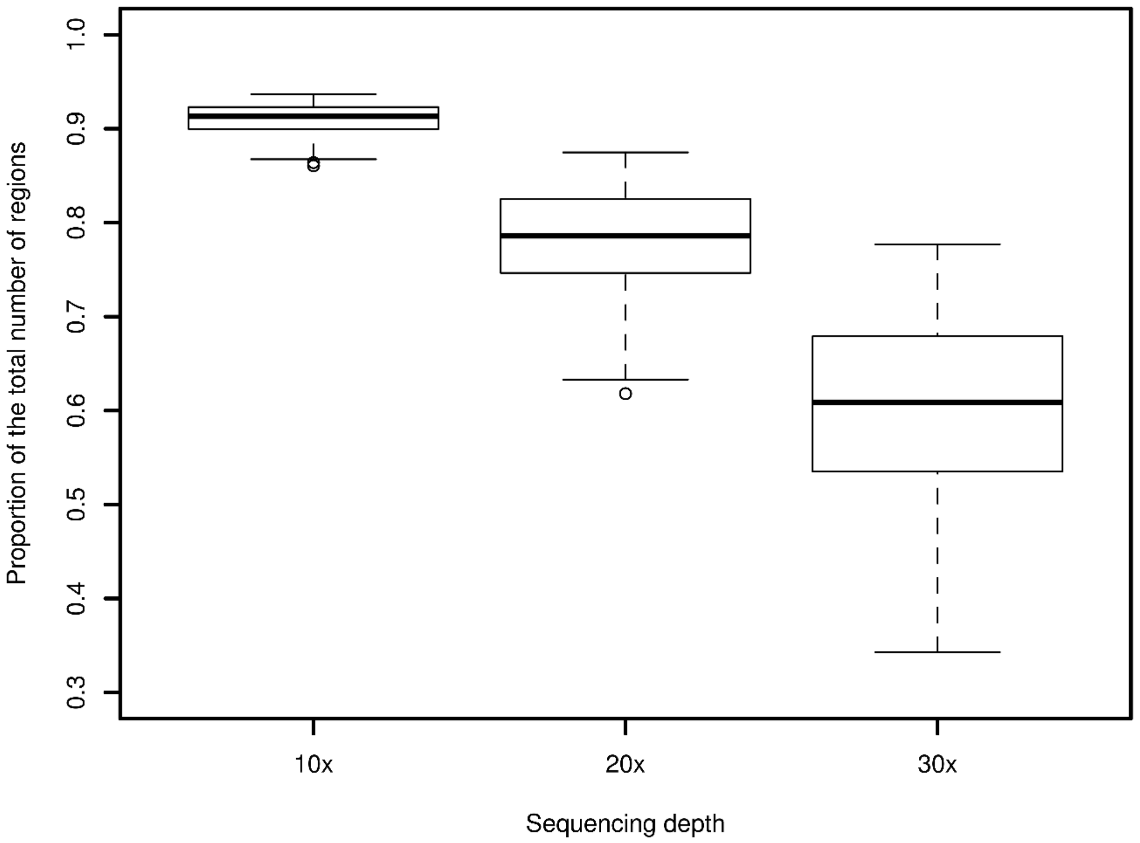

Fig. S4. Comparison of GC content (A), region size (B), repeat content (C) and ambiguous nucleotide content (D) for regions that are consistently sequenced at least 10x for all samples ("consistent") relative to regions that never reach a sequencing depth of 10x ("bad") and regions that have a varying sequencing depth ("variably"). Also see Table S5 for a numerical summary.

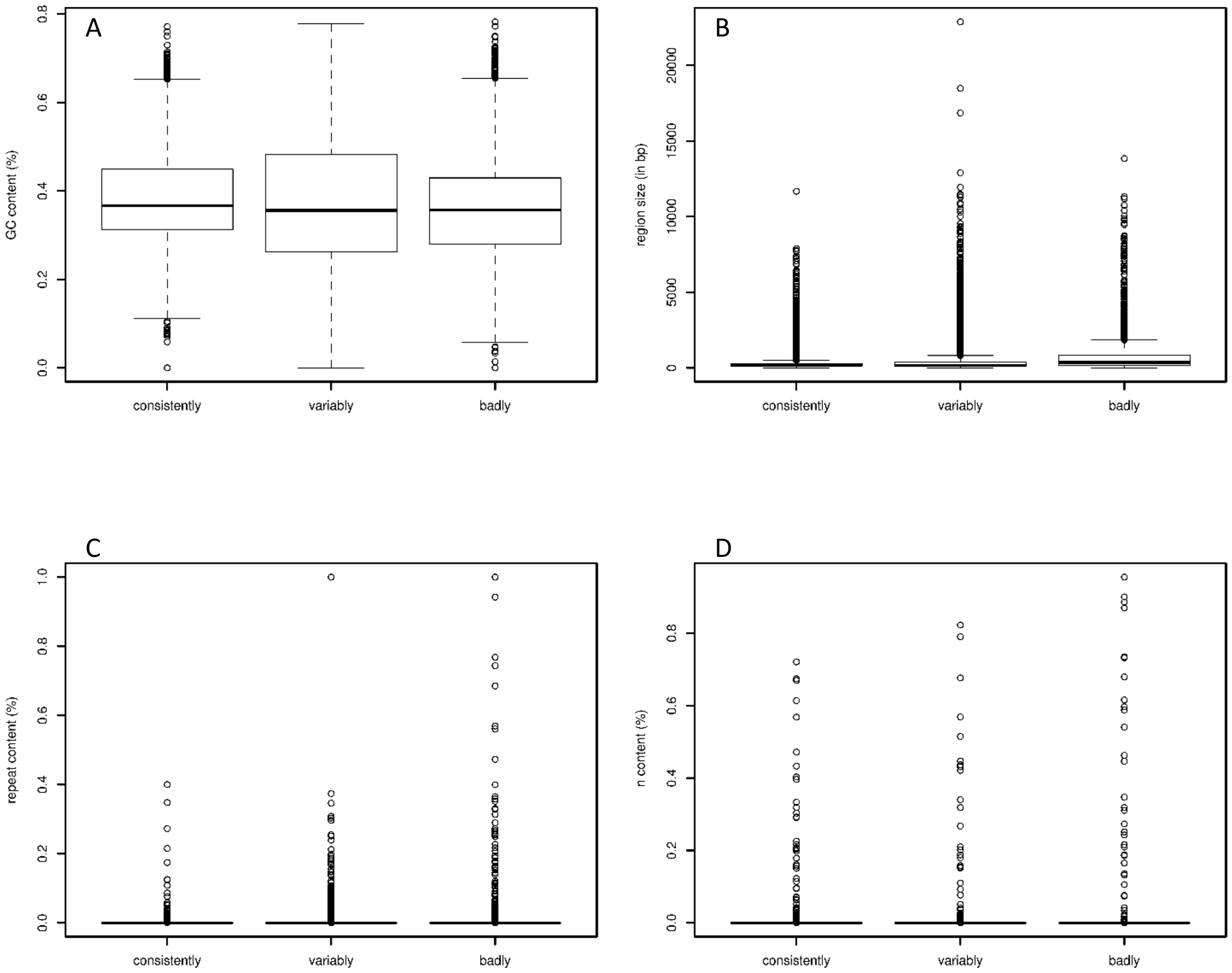
A
GC content (%)
B
region size (in bp)
C
repeat content (%)
D
n content (%)
consistently
variably
badly

Fig. S5. Distribution of distance between subsequent variants. Only those variants that passed the filters were used (see methods section). Distances are expressed in bp**.**

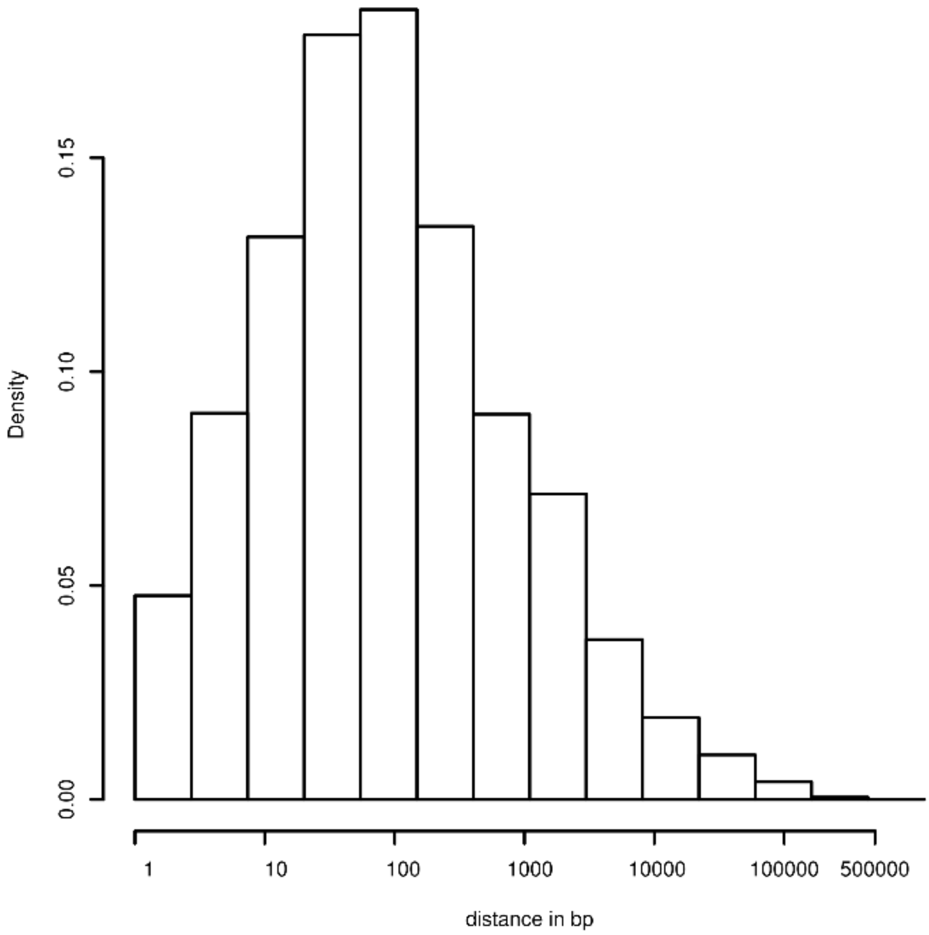

Fig. S6. Manhattan plot. The p-values were obtained using a Fisher exact test. The dashed line depicts the genome-wide significance threshold (corresponding to a Bonferroni-corrected p-value equal to 0.05/140,151 variants). The top 5 most significant variables were located on the two scaffolds plotted on the graph.

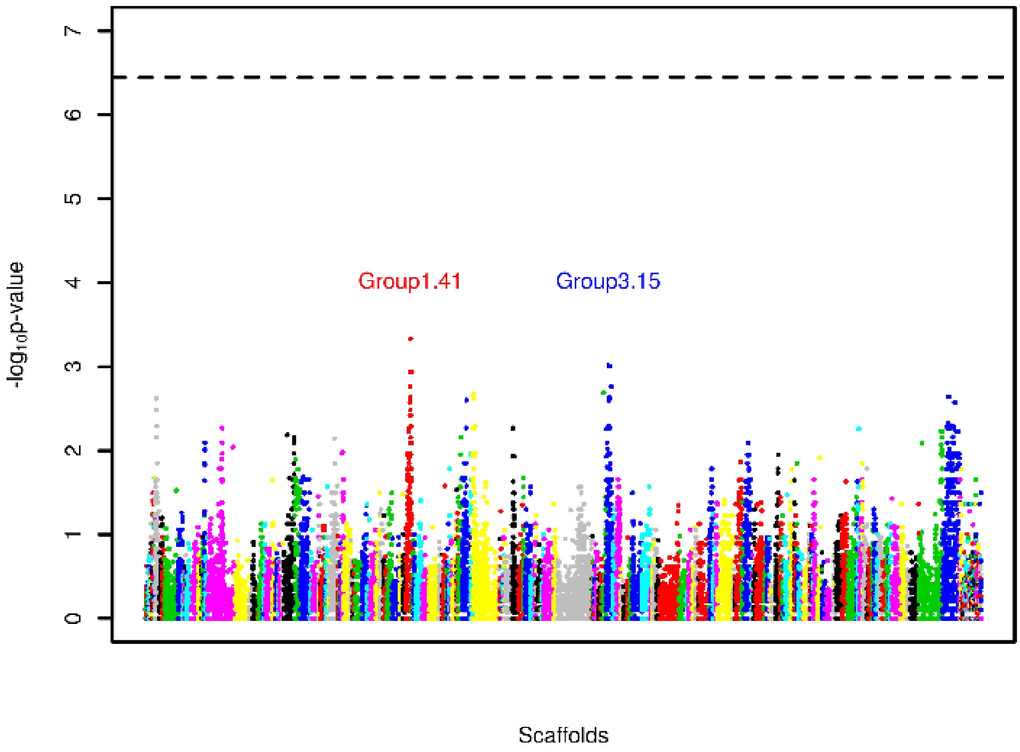

Table S1. *Varroa* reproductive success found in the F2 drone brood of the different genetic stocks. Numbers in the first column correspond with the origin of the genetic stock as also given in Fig. 1 (i.e. numbers 1-4 were *Varroa destructor*-resistant colonies; number 5 the *Varroa*-sensitive control: 1. Østlanded Region, Norway; 2. Amsterdam Water Dunes, The Netherlands; 3. Kapellen, Belgium; 4. Toulouse, France; 5. Ghent, Belgium). A Fisher exact test was conducted to compare the frequency of reproductive and non-reproductive mites in colony one to four relative to the control population of colony five. Significance was set at $\alpha \leq 0.05/4$ (with Bonferroni correction for multiple testing).

Origin	Total number of drone brood cells with mites	Drone brood cells with reproductive mites	Drone brood cells with non-reproductive mites	Percentage non-reproductive mites	Fisher Exact test p-value
1	139	95	44	31.6	0.31
2	69	34	35	51	0.01
3	28	24	4	14	0.71
4	24	15	9	37.5	0.20
5	21	17	4	19	reference

Table S2. Overview of the different microsatellite loci.

Loci	Repeat	Label	Range	Forward primer	Reverse primer	Reference
VD001	$(GA)_{10}(GA)_{6}(GA)_{5}$	VIC	170	CCCGCGAACGAAATAAATAGAG	AGCCACCTACGGTGCTCG	[2]
VD015	$(GT)_{14}$	VIC	164	GCGCAAACTTAACGCTCG	TCAAGCCAGAGTGCTGCAG	[2]
VD112	$(TC)_{16}(AC)_{8}(TC)_{4}$	FAM	160	TAACTATGGCCTAGCGACGG	CGTCGCTCATTATGGAACG	[2]
VD114	$(CT)_{14}$	PET	241	GCAGATTAGGAAGAATAAGCCG	CTCATACGACACTTGCCATAGG	[2]
VD146	$(AC)_{10}$	NED	188-200	TGGCTCATGCATTATCGTTG	AGCGTTTGGAGAGTGAGAAATAC	[2]
VD151	$(AC)_{7}GT(TA)_{4}$	NED	133	CGTTCAAGTATGCATACACACAC	CTAGGCATATTGGGCACG	[2]
VD163	$(CA)_{10}$	FAM	130-132	CAAGAGTCGGATTTGGCGC	TAGTATGCTTCTATATATCTCTGAGTTTTTAT	[2]
VD305	na	FAM	149	ACGGTATCCACTGCGTGACT	ATCATCAGTTGTTGCTCACCAC	[3]
VD306	na	NED	132	TGGTTGATGACCAGAGTTTACG	CTCGTCCTTGTTGCTATTGTTG	[3]
VD307	na	VIC	182-184	CTAATACCACCAAATGCTTCCG	CACAACAACAACAACAACAACG	[3]
VJ275	$(TC)_{23}$	NED	216-234	CGTCATACGTCTTTTAACTATCAGAG	GTTCGCAGGCGTAAGAACAT	[4]
VJ292	$(CT)_{18}CC(CT)_{5}$	FAM	253-255	TGAAAGCTGGTGTGAGTTACG	GCCCCTGACAACATGAACTT	[4]
VJ294	$(AG)_{23}$	VIC	190-192	GCTTCGTTCGGTCTTTTGTC	GCCTGACAGAATAGGCAAGC	[4]

Table S3. Genetic characterization of the *Varroa* mites from each studied colony. With *n* = number of mites genotyped, *N*e = effective number of alleles, *H*o = observed heterozygosity, *H*e = expected heterozygosity and *F*is = inbreeding coefficient. Numbers in the first column refer to the origin of the genetic stock as given in Fig. 1, followed by information of the reproductive success of the examined mother mite: NR = non-reproductive; R = reproductive. SE = standard error.

		*N*e		*H*o		*H*e		F_{is}	
Colony	*n*	Mean	SE	Mean	SE	Mean	SE	Mean	SE
5: NR	4	1.182	0.098	0.077	0.044	0.101	0.054	0.200	0.128
5: R	4	1.236	0.140	0.000	0.000	0.111	0.060	1.000	0.000
3: NR	4	1.145	0.098	0.019	0.019	0.075	0.050	0.733	0.105
3: R	4	1.328	0.141	0.000	0.000	0.169	0.064	1.000	0.000
2: NR	10	1.502	0.249	0.015	0.015	0.186	0.077	0.943	0.035
2: R	10	1.232	0.119	0.008	0.008	0.123	0.058	0.928	0.040
1: NR	10	1.299	0.186	0.008	0.008	0.125	0.064	0.737	0.146
1: R	10	1.384	0.219	0.031	0.024	0.147	0.071	0.765	0.106
Mean	8	1.289	0.044	0.020	0.010	0.130	0.014	0.788	0.100

Table S4. Relation between realized sequencing depth and the proportion of target base pairs (% of target bp) and the proportion of entirely sequenced target regions (% of regions) for which this sequencing depth is reached for individual samples.

	Sequencing depth		
% of target bp	≥10x	≥20x	≥30x
Q1	97.4	93.7	86.1
Median	97.7	94.8	88.6
Q3	97.8	95.7	91.1
% of regions			
Q1	90.0	75.0	54.1
Median	91.3	78.6	60.9
Q3	92.3	82.4	67.8

Table S5. Comparison of GC content, region size, repeat content and ambiguous nucleotide content ("Ns") (D) for regions that are consistently sequenced at least 10x for all samples ("consistent") relative to regions that never reach a sequencing depth of 10x ("bad") and regions that have a varying sequencing depth ("variably"). Also see Fig. S3 for a graphical representation.

Regions	Median GC (Q1-Q3)	Median region size (Q1 – Q3)	Repeats (%)	Ns (%)
Consistent	0.37 (0.31 – 0.45)	194 (133 – 288)	0.2%	0.2%
Variably	0.36 (0.26 – 0.48)	164 (92 – 382)	3.7%	0.6%
Bad	0.36 (0.28 – 0.43)	363 (148 – 826)	10.8%	3.2%

Table S6. Primers used to assess the fold enrichment.

Gene	Forward	Reverse	Reference
Actin	TGCCAACACTGTCCTTTCTG	AGAATTGACCCACCAATCCA	[5]
Enolase	GGTGATGAAGGTGGTTTTGC	GATGCAGCAACATCCATACC	[6]
GAPDH	GATGCACCCATGTTTGTTTG	TTTGCAGAAGGTGCATCAAC	[7]
eIF3-S8	TGAGTGTCTGCTATGGATTGC	TCGCGGCTCGTGGTA	[7]

Table S7. Primers used in the population study.

Gene	Forward	Reverse
Group1.41 (GB54921)	ACTTTTAGTCAGAAAATAGTAACGAG	TTCCGAAGAAGACTGGGAAA
Group10.23 (GB48382)	ATGGGAAACAGGAAAAGGTGGAGAGT	AGCACACCAACAGCCGAGTGA
Group15.14 (GB50526)	AGGAACGGGAGCGAGGATACGG	TCACCGAGGACGAGGAAGTAGCG
Group15.19 (GB50114)	TCAGCCGACGAGGAGGAGTCG	TCAGGGAGTCGAGGAACGTGTCG
Group3.15 (GB47018)	ACGCCTCGAATCCGCCTTTG	AGGAGTTGTTGTTTCTTTCGTGTCTCA
Group9.12 (GB53345)	GCGCGTTCATTGGTCTATTCGT	TCAGCATCGTCCTGGCGTAG
Group9.12 (GB53340)	ATCAGGCGTTCGAGGCTGAGA	AGGATGTTGACCGAGGCCAGA

The two variants at Group1.41 were genotyped with the same primer pair.

References

1. Earl, D. A. & vonHoldt, B. M. STRUCTURE HARVESTER: A website and program for visualizing STRUCTURE output and implementing the Evanno method. *Conserv. Genet. Resour.* **4,** 359–361 (2012).

2. Solignac, M. *et al.* Characterization of microsatellite markers for the apicultural pest Varroa destructor (Acari: Varroidae) and its relatives. *Mol. Ecol. Notes* **3,** 556–559 (2003).

3. Cornman, S. R. *et al.* Genomic survey of the ectoparasitic mite Varroa destructor, a major pest of the honey bee Apis mellifera. *BMC Genomics* **11,** 602 (2010).

4. Evans, J. D. Microsatellite loci in the honey bee parasitic mite Varroa jacobsoni. *Mol. Ecol.* **9,** 1436–1438 (2000).

5. Cunha, A. D., Nascimento, A. M., Guidugli, K. R., Simões, Z. L. P. & Bitondi, M. M. G.

Molecular cloning and expression of a hexamerin cDNA from the honey bee, Apis mellifera. *J. Insect Physiol.* **51,** 1135–1147 (2005).

6. Scharlaken, B. *et al.* Reference Gene Selection for Insect Expression Studies Using Quantitative Real-Time PCR: The Head of the Honeybee, *Apis mellifera* , After a Bacterial Challenge. *J. Insect Sci.* **8,** 1–10 (2008).

7. Fischer, P. & Grozinger, C. M. Pheromonal regulation of starvation resistance in honey bee workers (Apis mellifera). *Naturwissenschaften* **95,** 723–729 (2008).
